# Loss of RBPj in Postnatal Excitatory Neurons Does Not Cause Neurodegeneration or Memory Impairments in Aged Mice

**DOI:** 10.1371/journal.pone.0048180

**Published:** 2012-10-26

**Authors:** Chihiro Sato, Mustafa Turkoz, Joshua T. Dearborn, David F. Wozniak, Raphael Kopan, Matthew R. Hass

**Affiliations:** 1 Department of Developmental Biology, Washington University School of Medicine, St. Louis, Missouri, United States of America; 2 Department of Psychiatry, Washington University School of Medicine, St. Louis, Missouri, United States of America; Massachusetts General Hospital, United States of America

## Abstract

Previous studies suggest that loss of γ-secretase activity in postnatal mouse brains causes age-dependent memory impairment and neurodegeneration. Due to the diverse array of γ-secretase substrates, it remains to be demonstrated whether loss of cleavage of any specific substrate(s) is responsible for these defects. The bulk of the phenotypes observed in mammals deficient for γ-secretase or exposed to γ-secretase inhibitors are caused by the loss of Notch receptor proteolysis. Accordingly, inhibition of Notch signaling is the main cause for untoward effects for γ-secretase inhibitors as therapeutics for Alzheimer’s disease. Therefore, we wished to determine if loss of canonical Notch signaling is responsible for the age-dependent neurodegeneration observed upon γ-secrectase deficiency in the mouse brain. We generated postnatal forebrain-specific RBPj conditional knockout (cKO) mice using the CamKII-Cre driver and examined behavior and brain pathology in 12–18 month old animals. Since all four mammalian Notch receptor homologues signal via this DNA binding protein, these mice lack canonical Notch signaling. We found that loss of RBPj in mature excitatory neurons was well tolerated, with no evidence for neurodegeneration or of learning and memory impairment in mice aged up to 18 months. The only phenotypic deficit we observed in the RBPj-deficient mice was a subtle abnormality in olfactory preferences, particularly in females. We conclude that the loss of canonical Notch signaling through the four receptors is not responsible for age-dependent neurodegeneration or learning and memory deficits seen in γ-secretase deficient mice.

## Introduction

Alzheimer’s Disease (AD) is an incurable progressive neurodegenerative disease characterized by synaptic dysfunction and subsequent neurodegeneration, a hallmark of which are aggregates of amyloid β (Aβ) peptide (for a review see [1]). The Aβ peptide is a fragment of the amyloid precursor protein (APP) generated by two proteases: β-secretase and γ-secretase. The majority of mutations causing familial AD (FAD) are attributed to APP (the substrate), or to the catalytic subunits of the γ-secretase, Presenilin (PS) 1 and 2, indicating a central pathogenic role for the Aβ peptide in AD. This, and additional early observations, raised the hope that γ-secretase inhibition may offer a therapeutic strategy for delaying the onset of AD. However, reduced signaling through a γ-secretase dependent receptor, Notch, has a negative impact on memory and learning [2], [3], [4], [5], [6], [7], raising the concern that loss of Notch signaling associated with γ-secretase inhibition will exacerbate, rather than ameliorate, dementia. More recent studies supported that prediction by demonstrating that postnatal ablation of γ-secretase in the brain caused neurodegeneration and impairment of spatial memory and contextual fear conditioning in aged mice [8], [9]. Together, these results suggested that an inhibition of γ-secretase may worsen the symptoms of AD, an interpretation that was further supported when participants in a phase III trial for Eli Lilly’s γ-secretase inhibitor (GSI), semagacestat, reported reduced cognitive performance [10].

However, when haploinsufficiency of γ-secretase components was identified in human kindred suffering from the inflammatory skin disease hidradenitis suppurativa (HS) [11], [12], [13], there was no indication of cognitive deficits in these patients. γ-secretase mutations were seen in three of the four proteins: Nicastrin (Nct), Pen-2, and PS, which are all required for efficient proteolytic activity. Instead of cognitive problems, HS patients [14] and semagacestat-treated patients all experienced an increased incidence of non-melanoma skin cancer. Similarly, mice with reduced levels of γ-secretase [15], [16] or Notch signaling [17], [18] are more prone to skin cancer, which implicates Notch signaling as the substrate responsible for the oncogenic effects in humans. These recent findings raise doubt regarding the role of Notch signaling in the observed cognitive decline in the γ-secretase-deficient mouse brain and upon γ-secretase inhibitor treatment for AD.

Notch receptors are one of the most intensively investigated γ-secretase substrates and a major source for untoward effects in clinical trials of GSI. Given the ambiguity discussed above and data demonstrating roles for Notch signaling in synaptic plasticity (reviewed in [19]), we wished to test the consequences of removing all canonical Notch function from excitatory neurons in the adult brain. There are 4 mammalian Notch receptor homologues, and they all undergo proteolytic cleavage by the γ-secretase. This cleavage releases the Notch intracellular domain (NICD), which translocates to the nucleus where it interacts with its binding partner, RBPj, to regulate transcription (for a review see [20]). Therefore, we decided to test the consequences of Notch loss on cognitive functions by deleting two conditional RBPj alleles using the CamKII-Cre driver (RBPcKO). In the RBPcKO mice, all canonical Notch signaling (that is, RBPj-dependent) is postnatally ablated in the forebrain, yet immunohistological and behavioral analyses of RBPcKO mice at over 12 months revealed no evidence for age dependent neurodegeneration or learning and memory deficits. Interestingly, we observed that RBPcKO mice, particularly females, exhibited a subtle abnormality in olfactory preferences, suggesting a potential role for canonical Notch signaling in the maintenance of olfactory function in middle-aged and older female mice. The lack of obvious phenotypes in RBPcKO mice indicates that loss of canonical Notch signaling is not causing the age-dependent neurodegeneration seen in γ-secretase deficient brains, and suggests that RBPj mediated transcription or repression in excitatory neurons is not required for learning and memory.

## Results

### Generation of RBPcKO Mice Using CamKII-Cre

**Figure 1:**
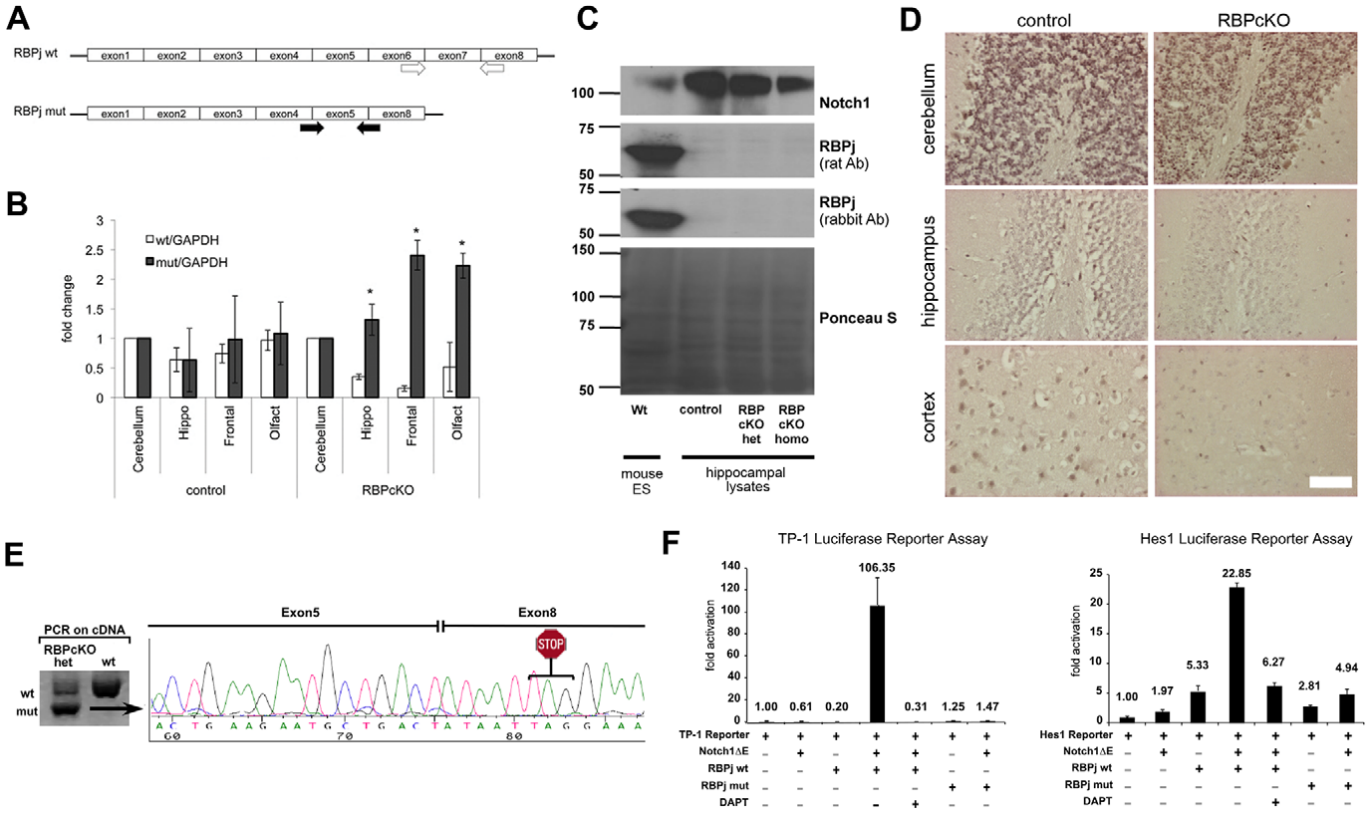
Generation of RBPcKO mice. (A) Strategy of detecting RBPj deletion using gene specific primers. Top: wild type RBPj (RBPj wt) and target primers (white arrows) that reside in exon 6 (forward) and exon 8 (reverse). Bottom: RBPj after deletion with Cre (RBPj mut) and target primers (black arrows) that bridge exons 4 and 5 (forward) and exons 5 and 8 (reverse). (B) mRNA levels of RBPj wt or RBPj mut in the cerebellum, hippocampus, frontal cortex, cerebellum and olfactory bulbs of control (RBPj f/f) or RBPcKO mice at 6 months. GAPDH was used as an internal control for qPCR. Fold changes are normalized to cerebellum where no gene deletion occurs under CamKII-Cre driver (*p<0.05). (C) Western blot analysis of RBPj in wild type mouse ES cells (left lane) and hippocampal lysates of control and RBPcKO mice (right 3 lanes). (D) DAB immunostaining of RBPj in the cerebellum, hippocampus, and frontal cortex of the control and RBPcKO mice. (E) Left: RBPj mut mRNA is not degraded by non-sense mediated decay in RBPcKO het mice. Right: Sequencing of the RBPj mut. Deletion of Exon 6 and 7 results in a frameshift and a stop codon after 2 amino acids into Exon8. (F) TP-1 and Hes1 Luciferase reporter assay. OT-11 cells are transfected with TP-1 or Hes1 reporters in addition to active Notch (Notch1ΔE), RBPj wt or RBPj mut, and +/− DAPT (γ-secretase inhibitor).

Previous studies have observed that CamKII-Cre mediated loss of PS or Nct in post-mitotic excitatory neocortical neurons results in adult onset neurodegeneration [8], [9]. In order to determine whether loss of canonical Notch signaling underlies this neurodegeneration, we crossed RBPj flox/flox (f/f) mice, in which the exons 6 and 7 are flanked by two loxP sites ([21], Figure 1A), with CamKII-Cre transgenic mice that express Cre recombinase in excitatory neurons in the postnatal forebrain. These mice were viable and visually indistinguishable from the littermate controls.

To confirm the deletion of RBPj in the hippocampus and frontal cortex where CamKII-Cre is expressed, we performed qRT-PCR to determine the level of RBPj mRNA in the mice brains. Intact RBPj f/f will be maintained in glia, vasculature, and non-excitatory neurons in RBPcKO mice. Thus, we designed primers that specifically differentiate wild-type RBPj (RBPj wt, prior to Cre recombination and present in non-neuronal cells) from mutant RBPj (RBPj mut; generated by Cre in excitatory neurons) (Figure 1A). QRT-PCR suggested that, in RBPcKO mice, mRNA levels of RBPj wt decreased by 65% (p = 0.037), 85% (p = 0.007), and 48% (p = 0.077) in the hippocampus, frontal cortex, and olfactory bulb, respectively (Figure 1B). Conversely, the mRNA level of RBPj mut increased in the hippocampus, frontal cortex and olfactory bulb (132% (p = 0.310), 231% (p = 0.052), and 222% (p = 0.040) respectively). There was no significant change in mRNA levels of either RBPj wt or RBPj mut in the control littermates. In agreement with the lack of RBPj expression indicated by the Allen Brain Atlas, Western blot analyses using two independent antibodies for RBPj find only minimal expression of RBPj in adult mouse brain extracts (Figure 1C). Nevertheless, using immunostaining with DAB amplification we were able to observe loss of RBPj immunoreactivity in the hippocampus and frontal cortex but not the cerebellum, as expected (Figure 1D).

Cre-mediated recombination of the floxed RBPj allele generates an RNA that could encode for a truncated RBPj protein (RBPj mut. Figure 1E). We tested whether this protein could retain the ability to transmit canonical Notch signaling. Transfection of OT-11 (RBPj-deficient) cells [22] with RBPj wt rescued the ability of an activated Notch allele to induce the reporters, TP-1 or Hes1; the RBPj mut could not (Figure 1F). Furthermore, a VP16-fusions to RBPj mut was still unable to activate the reporters (data not shown), consistent with the removal of most of the DNA binding domain [21]. This confirms that Cre-mediated loss of RBPj in the excitatory neurons would eliminate all canonical Notch signaling.

### Normal Brain Morphology in RBPcKO Mice at 12 Months

**Figure 2:**
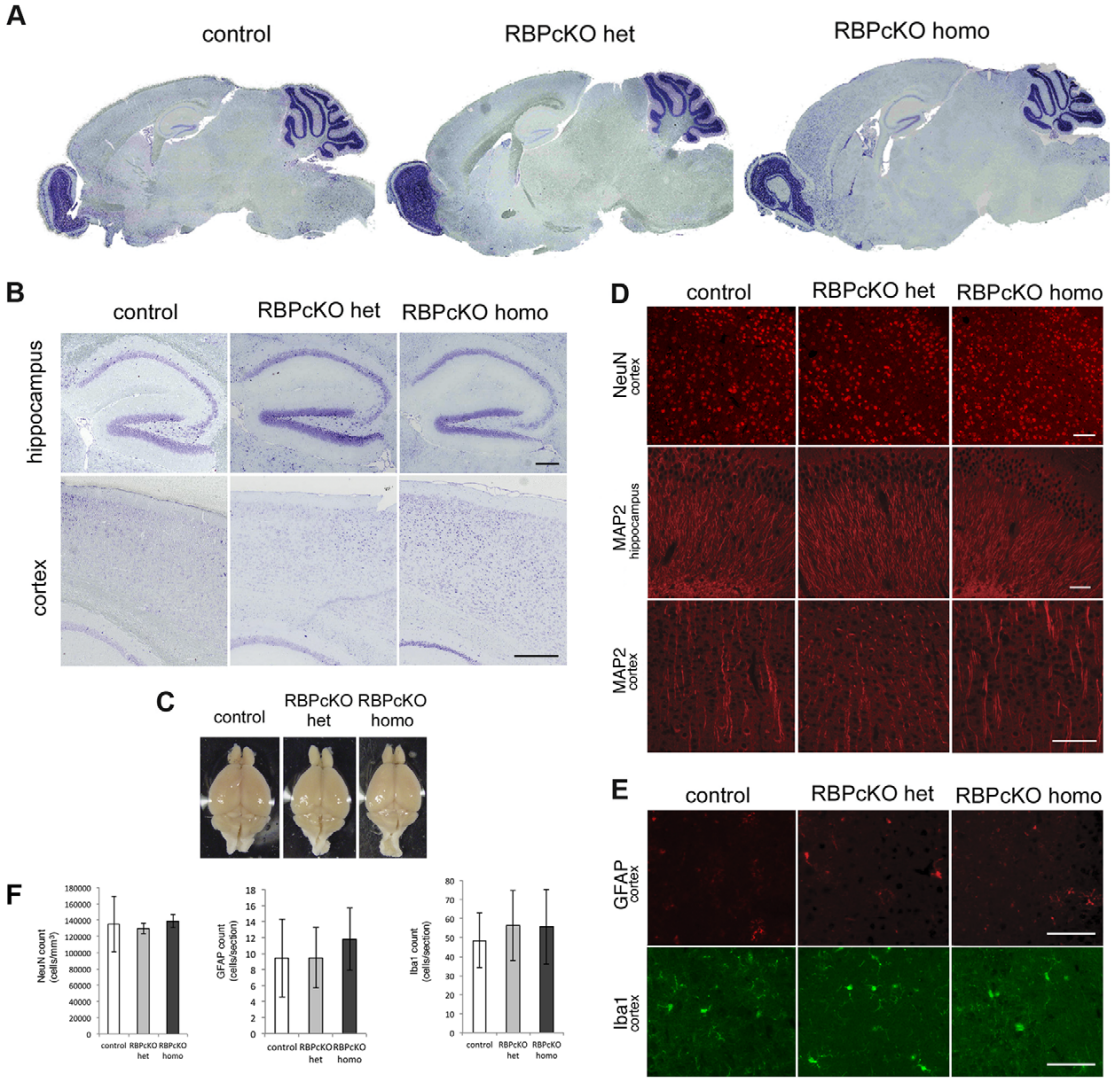
Normal brain morphology in RBPcKO mice at over 12 months of age. (A) Nissl staining of sagittal sections of control and RBPcKO mice brains. (B) Representative higher magnification view of the hippocampus and the cortex of each genotype (Scale bars = 200 µm). (C) Pictures of the brains from control and RBPcKO mice after dissection. (D) NeuN staining in of he three genotypes in the cortex (top) and MAP2 immunostaining in the hippocampus and the cortex (Middle and bottom. Scale bar = 100 µm). (E) GFAP (top) and Iba1 (bottom) immunostaining of the three genotypes in the cortex (Scale bar = 100 µm). (F) Number of neurons (NeuN positive), astroglia (GFAP positive), and activated microglia (Iba1 positive) in the cortex of each genotype.

We then investigated whether the deletion of RBPj has any effects on brain morphology in brain sections from RBPcKO mice 12 to 18 months of age. Nissl staining of the brain sections from RBPcKO mice indicated normal brain architecture with no differences in the thickness of the cortex (Figure 2A, 2B). We could detect no difference in either the volume or the weight of the brain between the RBPcKO mice and the age-matched control littermates (Figure 2C and data not shown). Quantification of neuronal number by immunostaining for NeuN showed no statistical differences between the different genotypes (Figure 2D top, 2F left). Immunohistochemical analyses indicated that there was no change in the MAP2 reactivity in the neocortex and hippocampus between the three genotypes, suggesting normal neuronal structure in the RBPcKO mice (Figure 2D middle, bottom). These results together suggest normal brain cytoarchitecture and morphology in RBPcKO mice at 12 to 18 months.

We further examined if RBPcKO mice had any indications of severe inflammation and gliosis that often accompany neurodegeneration. Using optical dissector method, we detected no significant differences in the number of astroglia or activated microglia detected by GFAP and Iba1, respectively, between the three genotypes throughout multiple brain regions (Figure 2D middle and bottom, 2F middle and right).

### Spatial and Contextual Fear Memory is not Impaired in RBPcKO Mice at 12 Months

Before testing learning and memory capabilities of the mice, we performed basic locomotor and sensorimotor tests to determine if there were functional deficits in the RBPcKO mice that might compromise their performance on the cognitive tests. No significant effects involving genotype were found, thus documenting that the groups did not differ significantly in ambulation or vertical rearing or on any of the indices of emotionality (ANOVAs conducted on the 1-h locomotor activity variables, data not shown). Similarly, there were no significant effects involving genotype for any of the measures within the sensorimotor battery, suggesting that these functions were intact in RBPcKO mice.

**Figure 3:**
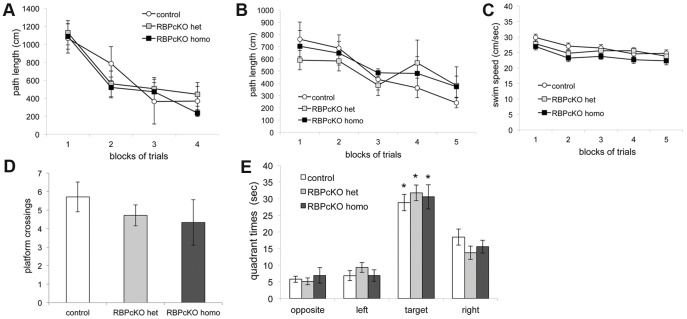
Lack of spatial learning and memory impairments in RBPcKO mice at 12–18 months of age. (A) RBPcKO homozygous (homo) mice performed similarly to WT control and RBPcKO heterozygous (het) groups in terms of escape path length during cued trials in the water maze. (B–C) No significant differences were observed between the RBPcKO homo group and the WT control or RBPcKO mice during the place (spatial learning) trials with regard to path length (B) or swimming speed (C). (D–E) No significant differences were found in retention performance between RBPcKO homo mice and the WT control and RBPcKO het groups concerning platform crossings (D) or spatial bias (E) during the probe trial. Each of the genotypes exhibited spatial bias for the target quadrant where the platform had been located by spending significantly more time in the target quadrant, compared to the time spent in each of the other quadrants (*p<0.008).

To assess the effects of loss of RBPj in hippocampus-dependent spatial learning and memory, RBPcKO mice and control littermates at 12–18 months of age were evaluated on the Morris Water Maze (MWM) tasks. The mice were first tested on the cued (visible platform) trials to document that the RBPcKO mice did not have any non-associative (visual, sensorimotor, or motivational) disturbances that would affect their subsequent spatial learning/memory performance. A rmANOVA conducted on the escape path length data from the cued condition (Figure 3A) did not reveal any significant overall effects involving genotype, and all groups exhibited similar learning curves, suggesting that there were no differences in cued learning capabilities. The same results were found for escape latency (not shown). Although the RBPcKO homozygous mice tended to swim more slowly than the WT littermate controls, the rmANOVA did not reveal any significant effects involving genotype (not shown). In summary, the cued trials data supported the results from the 1-h locomotor activity test and the sensorimotor battery in that there was no evidence suggesting that the RBPcKO mice had any non-associative disturbances that were likely to affect their spatial learning/memory performance.

A rmANOVA conducted on the path length data from the place trials (spatial learning; Figure 3B) yielded a significant effect of blocks of trials, [F(4,84) = 7.91, p = 0.0001], indicating that the acquisition performance of the groups improved across the test period and that place learning had occurred. However, there were no significant effects involving genotype, suggesting that the RBPcKO mice were not impaired during the place condition. Similarly, no differences were found in escape latencies between the RBPcKO group and the WT control mice (not shown). Again, the RBPcKO homozygous mice tended to swim more slowly than the WT control group, but no significant effects of genotype were found following a rmANOVA conducted on these data (Figure 3C). The results from the post-training probe trial were consistent with those from acquisition training in the place condition in that no differences were observed between groups with regard to number of platform crossings over the location of where the platform had been located (Figure 3D) or time spent in the target quadrant (Figure 3E). In addition, all groups showed spatial bias for the target quadrant (Figure 3E) in that each group spent significantly more time in the target quadrant compared to the times spent in each of the other quadrants (p<0.008).

**Figure 4:**
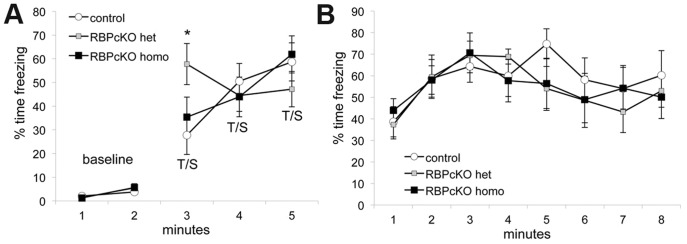
No contextual fear conditioning deficits were observed in RBPcKO mice. (A) RBPcKO homo mice showed levels of baseline freezing (% time spent freezing) which were similar to those observed in WT control and RBPcKO het groups during the first 2 min of testing on day 1 (A-left panel). The results of ANOVA and pair-wise comparisons conducted on the freezing data during tone-shock (t/s) training on day 1 (A-right panel; mins 3–5) indicated that differences in freezing were observed only during minute 3 when the RBPcKO het mice froze more often than WT controls (*p = 0.02), and most of this was due to differences between the males of the two groups (p = 0.0006; not shown). No significant differences in freezing levels were observed between groups during minutes 4–5 of t/s training. (B) No significant differences in freezing levels were observed between the genotypes during testing on day 2, showing that the RBPcKO homo mice were not impaired in terms of contextual fear conditioning.

Nonspatial learning and memory functions were also examined in the RBPcKO and WT control littermates by assessing their performance on the conditioned fear procedure. On day 1, all three groups of mice exhibited similar levels of baseline freezing during the first two minutes in the training chamber, and this was confirmed by the results of an rmANOVA that failed to reveal any significant overall effects involving genotype (Figure 4A). During the first tone-shock (t/s) pairing, the RBPcKO heterozygous mice showed a heightened degree of freezing compared to the other two groups, and this was confirmed by a significant genotype by minute interaction, [F(4,36) = 7.55, p = 0.0002], and by a genotype by minute by sex interaction, [F(4,36) = 3.76, p = 0.012]. Subsequent pair-wise comparisons showed that these effects were mostly due to differences between the male WT control and male RBPcKO heterozygous (p = 0.0006) and male RBPcKO homozygous (p = 0.036) mice (data not shown), while no differences were found between the groups of female mice. This performance difference was a short-lived response in the RBPcKO heterozygous mice, such that all three groups showed similar levels of freezing in response to the second and third t/s pairings (Figure 4A). All three groups of mice exhibited similar freezing levels during the contextual fear test conducted on day 2 (Figure 4B), and this was confirmed by the absence of any significant overall effects involving genotype. We also conducted an additional analysis comparing the average freezing level during the baseline testing on day 1 with the average freezing levels observed during the first two minutes of the contextual fear test. As expected, no differences were observed between the groups at either test period, and each group showed very significant elevations in freezing during the initial phases of the contextual fear testing (not shown) compared to baseline (p<0.00005), thus suggesting that the RBPcKO mice were unimpaired with regard to conditioning to the contextual cues in the training chamber.

No significant effects involving genotype were found following ANOVAs conducted on the altered context baseline freezing data collected during the first two minutes after the mice were placed into the other chamber containing different cues on day 3 (data not shown). Similarly, no significant effects involving genotype were revealed following analysis of the freezing data from the subsequent 8 min when the tone was presented during auditory cue testing (not shown). Firm conclusions are difficult to draw from the auditory cue data since mice may have hearing impairments during these ages [23], [24]; additional control groups are needed to distinguish between conditioning to the tone and sensitization. No differences in shock sensitivities were found among the groups with regard to intensities that were required to elicit flinching or vocalization. Collectively, the results from our cognitive tests suggest that spatial learning, memory and contextual fear conditioning are not impaired in RBPcKO mice that are 12–18 months old.

### Impaired Olfactory Function in Female RBPcKO Mice at 12 Months

**Figure 5:**
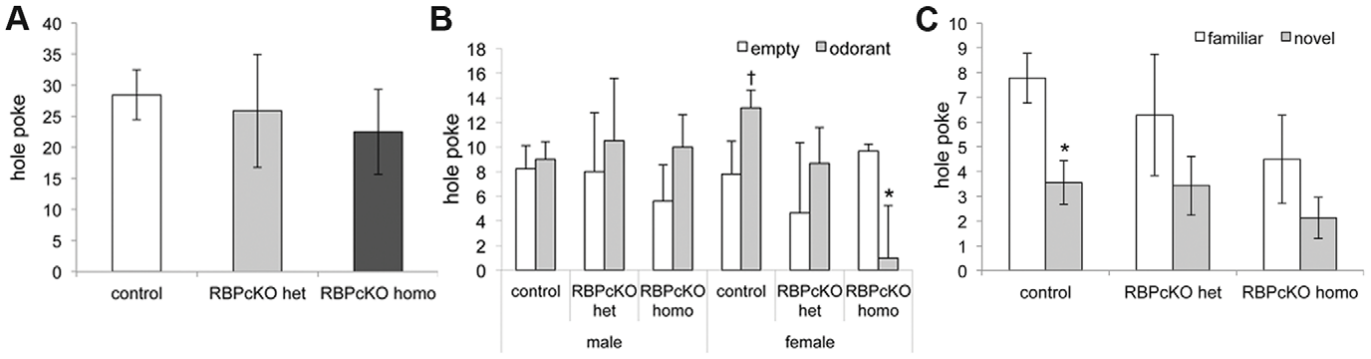
RBPcKO homozygous female mice exhibited atypical olfactory preferences. (A) The three groups showed similar frequencies of total hole pokes, thus establishing that there were no differences in this type of exploratory activity which would affect subsequent olfactory preference behaviors. (B) An ANOVA conducted on the poke frequencies into empty and odorant-containing corner holes revealed a significant genotype by sex by hole interaction (p = 0.006), and subsequent comparisons showed that the female RBPcKO homo mice had a significant preference (beyond Bonferroni correction) for the empty versus the odorant-containing holes (*p = 0.008), while other groups, such as the WT females, preferred (†p = 0.028), or at least tended to prefer, the odorant-containing holes. (C) Planned comparisons revealed that only WT control mice showed a significant preference for the familiar (bedding) odor versus the novel (coconut) odor (*p = 0.0025), although the RBPcKO groups showed a tendency toward the same preference.

A previous study using adenovirus Cre-mediated deletion of RBPj in the subventricular zone resulted in a reduction in the number of mature granule cells in the olfactory bulb [25]. To examine certain aspects of olfactory function in RBPcKO mice, RBPcKO mice and control littermates at 12–18 months of age were evaluated on the holeboard exploratory/olfactory preference test [26]. In the holeboard test, mice are placed in a test chamber containing four corner holes and four side holes and the number of times that a mouse pokes its head into a hole (at a certain depth) and the duration of pokes are recorded to quantify exploratory behavior and olfactory preference. General exploratory behavior is assessed by quantifying the number of total hole pokes, as well as differentiating the number of pokes made into the corner and side holes. The RBPcKO and control mice exhibited similar levels of poking on all three of these variables (Figure 5A and data not shown), indicating that they did not differ from one another in terms of their general hole poking proclivities. To assess behaviors related to olfactory preference, we restricted analysis of hole poking to the corner holes where two opposing corner holes contained odorants, and the other two opposing corner holes were empty. With regard to the odorants, one was familiar (bedding) and one was novel (coconut). Mice tend to poke more often into odorant-containing versus empty corner holes, but show a robust preference for poking into holes containing the familiar scent of (fresh) bedding used in their home cages over a novel odorant [26], [27].

To evaluate olfactory preferences, we conducted planned comparisons on the number of hole pokes made into the odorant-containing versus empty corner holes, as well as pokes into holes containing familiar or novel odorants. Results from these comparisons showed that none of the groups significantly preferred the odorant-containing versus the empty corner holes, although the WT control group tended to do so (p = 0.086; data not shown). However, an ANOVA conducted on these data revealed a significant genotype by sex by hole interaction, [F(2,18) = 6.84, p = 0.006], suggesting sex-dependent effects across groups. Inspection of these data indicated that males and females of each genotype showed at least a tendency to prefer the odorant-containing versus the empty corner hole except for the female RBPcKO homozygous mice, which exhibited the opposite preference (Figure 5B). Subsequent within-subjects comparisons showed that female WT control mice exhibited some preference for the odorant-containing versus the empty corner holes (p = 0.028), while WT males did not, and that the male and female RBPcKO heterozygous mice and male RBPcKO homozygous mice each showed non-significant trends for preferring the odorant-containing corner holes. In contrast, the female RBPcKO homozygous mice exhibited a robust preference for the empty corner holes over the odorant-containing holes, which was significant beyond Bonferroni corrected levels (p = 0.0081).

Planned comparisons conducted on poke frequencies into familiar and novel odorant-containing holes indicated that the WT control mice exhibited the expected robust preference for the familiar odorant, [F(1,21) = 11.78, p = 0.0025], while non-significant trends for this preference were shown by the RBPcKO heterozygous (p = 0.064) and RBPcKO homozygous groups (Figure 5C). An ANOVA conducted on these data did not reveal any sex-dependent effects. It should be noted, however, that the female RBPcKO homozygous mice tended to poke more frequently into the novel odorant-containing hole, unlike all of the other groups, but they poked so infrequently into the odorant-containing holes that it was not possible for the analyses to accurately reflect their preferences for the familiar versus novel odors. We also analyzed the average duration of hole pokes with regard to total pokes, pokes into empty holes and pokes into odorant-containing holes, and rmANOVAs conducted on these variables did not reveal any significant overall effects involving genotype (data not shown). In summary, our olfactory preference data suggest that RBPcKO mice do not have the same preferences as WT littermate controls, and that female RBPcKO homozygous mice may have significant abnormal preferences, although the mechanistic basis for this latter possibility needs to be determined by additional studies.

## Discussion

### Role of Canonical Notch Signaling in Neurodegeneration and Memory

Previous studies identified age-dependent neurodegeneration when either PS or Nct were removed from excitatory neurons in postnatal forebrain [8], [9]. This indicated that neurodegeneration could be caused by loss of γ-secretase activity but did not establish a molecular mechanism. Assigning a mechanism is complicated due to the presence of many γ-secretase substrates involved in a multitude of different signaling pathways [28]. This study is focused on the extent to which loss of Notch signaling contributes to the phenotypes seen in γ-secretase deficient mice.

Hints that Notch signaling may be implicated in this phenotype abound in the literature. Reduced Notch activity was linked to poor cognitive performance in mice heterozygous for Notch1 or RBPj [5] or in mice with CamKII-Cre mediated deletion of Notch 1 [6]. Additionally, Notch signaling through Suppressor of Hairless (Drosophila homologue of RBPj) has been shown to be required for certain behavioral functions in postnatal flies [3], [4], [29]. Finally, reduced RBPj-dependent Notch activity is thought to mediate the untoward effects caused by γ-secretase deficiency or inhibition in most other tissues, such as the intestine, hematopoietic system, skin, and skeletal muscle (reviewed in [30]). Contrary to expectation, our results argue that cognitive decline in any of these models is unlikely to be reflective of losing canonical Notch function in excitatory neurons. This conclusion is supported by the recently reported absence of phenotype in adult mice brains lacking Notch1 and Notch2 [31]. Previously observed defects could be due to loss of Notch signaling in other cell types in the brain, such as inhibitory neurons or glia, to an RBPj-independent Notch function mediated at the synapse or at the cell body by Notch3 or Notch4 [31], or due to an alternative γ-secretase substrate. For instance, Notch signaling has been implicated in adult neurogenesis, inhibitory neuron diversification, and the generation of astrocytes [19], [32], [33], [34], [35], [36], [37].

We showed that aging RBPcKO mice lacking canonical Notch signaling in excitatory neurons of the forebrain were anatomically and behaviorally indistinguishable from their wild-type littermates. Although much larger cohorts may reveal subtle differences, this is in dramatic contrast to the severe deficits observed in similar size cohorts of aged PScDKO and NctcKO mice [8], [9], which included neurodegeneration and clear memory impairments at 12–18 months of age. Compared to the robust gliosis detected in aged PScDKO and NctcKO mice, no significant differences were observed in the total astroglia or activated microglia in RBPcKO cortex. Furthermore, we did not observe any differences between the three genotypes in the Morris Water Maze or in contextual fear conditioning; again, it is possible that a study of a larger cohort of RBPj-deficient mice may detect subtle memory deficits similar to that observed in mice deficient for Notch1 [6]. We conclude that the postnatal ablation of canonical Notch signaling is not responsible for the dramatic neurodegeneration or severe learning and memory impairments caused by γ-secretase deletion.

We propose three possible explanations for these observations. First, another substrate or a combination of substrates are responsible for the previously observed age- and γ-secretase-dependent neurodegeneration and memory impairments. Second, because loss of both Notch1 and Notch2 is tolerated with no evidence for neurodegeneration [31], Notch3 and 4 may be responsible for the neurodegeneration but through RBPj independent (non-canonical) Notch signaling pathway acting at the synapse or the cell body. Third, the phenotypes result from the loss of the “proteasome of the membrane” function of γ-secretase [38], [39]; this leads to the over-accumulation of transmembrane spanning C-terminal fragments from its myriad of substrates. In neurons, whose membrane to cytoplasm ratio is extreme, loss of this housekeeping function may be poorly tolerated, making neurons more susceptible to toxicity associated with increased protein content within the plasma membrane. Future experiments will be required to test which of these possibilities best explains the neurodegeneration in aging mice lacking γ-secretase.

### Role of Canonical Notch Signaling in Adult Olfaction

Interestingly, we observed that female RBPcKO mice at 12–18 months of age process certain olfactory stimuli differently compared to WT controls, suggesting that RBPj may mediate certain olfactory functions. This hypothesis is consistent with recent reports that Notch signaling may play a role in the generation of mature granule cells in the olfactory bulb [25] and in olfactory neuron diversification and function in *Drosophila* [40], [41]. However, it is unclear whether the differences in olfactory preference in the RBPcKO mice reflect sensory deficits or abnormal higher-order association disturbances involving the rewarding properties of odorants, which might underlie approach-avoidance behaviors. Although our olfactory preference results suggest that RBPj may be involved in some aspects of olfactory function, further studies involving more extensive olfactory preference testing are needed to provide explain the biological and mechanistic basis of these deficits and whether there are sex-dependent effects.

In summary, Notch signaling deficiency in excitatory neurons is not responsible for the age-dependent neurodegeneration and memory impairment observed in the absence of γ-secretase. In addition, excitatory neurons in the neocortex maintain normal function in the absence of canonical Notch signaling in the adult mouse brain.

## Materials and Methods

### Ethics Statement

Animal studies were approved by the Washington University Division of Comparative Medicine, protocol #20110027 and the mice were cared for following ICOC guidelines.

### Generation of RBPcKO Mice

All mouse lines used in this study have been previously described: RBPj f/f [42] and CamKII-Cre Tg mice [43]. Forebrain-specific RBPj conditional knockout mice (RBPcKO) were generated by crossing RBPj f/f with CamKII-Cre Tg mice. All three genotypic groups (RBPj f/f, CamKII-Cre RBPj f/+, CamKII-Cre RBPj f/f) were obtained from crossing CamKII-Cre x RBPj f/+ and RBPj f/f mice, and littermates were used for all experiments. These mice were generated in the C57BL6/CD1 mixed background. Genotyping was performed using the universal PCR genotyping protocol [44].

### Dissection

Mice were anesthetized and cardiac perfusion was conducted with phosphate-buffered saline (PBS) and 4% paraformaldehyde (PFA). For isolating different parts of the brain, after removing cerebellum and olfactory bulb, brain was cut sagittally in half and banana-shaped area encompassing the hippocampus was dissected out on ice. The frontal cortex was cut from the remaining tissue.

### Quantitative Real-Time PCR

mRNA was extracted from isolated cerebellum, hippocampus, frontal cortex, and olfactory bulb using RNeasy kit (Qiagen). Total RNA (1 ug) was treated with DNase I and reverse transcribed using the SuperScript First-Strand Synthesis kit (Invitrogen). PCR reactions in triplicate were performed in a total volume of 25 µl using SYBR Green PCR mastermix in a Step One Plus Real-time PCR system (Applied Biosystems) using 5 µl of diluted (1∶100) cDNA and gene-specific primers. Amplification was performed under the following conditions: 40 cycles of 95°C for 20 s and 60°C for 20 s. The forward primer for RBPj wt is located in exon 6 that is deleted in RBPcKO, and the reverse primer is located in exon 8. The forward primer for RBPj mut spans the junction between exon 4 and 5, and the reverse spans the novel junction between exons 5 and 8 that is generated upon Cre-mediated deletion of exon 6 and 7. We prepared mRNA from hippocampus, frontal cortex, cerebellum and olfactory bulb from 6 months old RBPcKO and control RBPj f/f mice. GAPDH was used as an internal control for PCR reaction, and the ratio of mRNA was normalized to that of cerebellum where deletion does not occur.

### Western Blot

Lysates were generated from various brain regions in RIPA-DOC, homogenized through sonication and equivalent amounts as determined by protein assays of the brain lysate and from mouse ES cells were loaded on reducing polyacrylamide gels. The lysates were probed with antibodies against total Notch1 (rabbit monoclonal #3608 (Cell Signaling)) and two independent antibodies for RBPj (rabbit polyclonal #5442 (Cell Signaling) and rat monoclonal T-6709 (Cosmo Bio)). Equivalent loading was confirmed by staining for Ponceau S.

### Notch Reporter Assay

RBPj wt and RBPj mut sequences were cloned into the HindIII and BamHI sites in 3x Flag CMV-7 vector (Sigma-Aldrich, St. Louis, MO). TP-1 and Hes1 luciferase reporters were previously published [45], [46]. OT11 cells (RBPj −/−, gift from Dr. Tasuku Honjo) were seeded at 50×10*3 cells/mL density in 0.5 mL/well (24well plate) in antibiotic free DMEM supplemented with 10% FBS. Following day, cells were transfected in Opti-MEM I reduced serum medium with 500 ng DNA containing (300 ng reporter vector, 10 ng Notch1ΔE (Notch1 lacking extracellular domain, i.e. active Notch), 100 ng RBPj wt (or RBPj mut) and appropriate amount of balance DNA) using Lipofectamine LTX and PLUS according to the manufacturer’s instructions. On day 3, media were changed. On day 4, cells were lysed and luciferase assays were performed as previously described [47]. For each reporter, fold stimulation was expressed relative to the activity of the empty vector control, which was normalized to a value of 1. All data points were obtained triplicate and error bars show s.d. The data reported in the figures are representative of minimum three independent experiments in each case.

### Behavioral Tests

A cohort of mice aged 12–18 months (RBPj f/f (control) n = 9 (M4, F5): CamKII-Cre RBPj f/+ (RBPcKO het) n = 7 (M4, F3): CamKII-Cre RBPj f/f (RBPcKO homo) n = 8 (M5, F3)) was evaluated through the use of several behavioral analyses, including a (1-h) locomotor activity/exploratory behavior test, a battery of sensorimotor measures, as well as the Morris water navigation, holeboard exploration/olfactory preference, and conditioned fear tests (Figure S1). Same cohort of mice was subjected to immunohistochemical and biochemical analyses after the behavioral analyses. All behavioral tests were conducted by an individual who was “blinded” with regard to the genotype of the mice being tested.

#### 1-h locomotor activity and sensorimotor battery

Locomotor activity was evaluated in all mice over a 1-h period using transparent (47.6×25.4×20.6 cm high) polystyrene enclosures and computerized photobeam instrumentation, as previously described [48]. General activity variables (total ambulations, rearings), along with measures of emotionality including time spent, distance traveled and entries made in a 33×11 cm central zone, were analyzed. All mice were also evaluated on a battery of sensorimotor tests to assess balance (ledge and platform), strength (inverted screen), coordination (pole and inclined screens) and initiation of movement (walking initiation), as previously described [48], [49].

#### Morris water navigation

Spatial learning and memory were evaluated in the Morris water maze using a computerized tracking system (ANY-maze, Stoelting Co., Wood Dale, IL), as previously described [48], [50]. Cued (visible platform, variable location) and place (submerged, hidden platform, constant location) trials were conducted, and escape path length, latency, and swimming speeds served as dependent variables. Mice first received cued trials to determine if non-associative dysfunctions (sensorimotor or visual disturbances or alterations in motivation) were likely to affect performance in subsequent place trials. The cued trials involved conducting 4 trials per day (60 s maximum per trial) for 2 consecutive days, with the platform being moved to a different location for each trial, using a 30-min inter-trial interval (ITI) and with very few distal spatial cues being present to limit spatial learning. Performance was analyzed across four blocks of trials (2 trials/block). Three days later, place trials were initiated to assess spatial learning where mice were required to learn the single location of a submerged platform in the presence of several salient distal spatial cues. During place trials, the mice received 2 blocks of 2 consecutive trials [60 s maximum for a trial; 30-s ITI (spent on platform)], with each block being separated by approximately 2 h and each mouse being released from a different quadrant for each trial. The place trials data were analyzed over five blocks of trials (4 trials/block) where each block represented the performance level for each of five consecutive days. A probe trial (60-sec maximum) was administered approximately 1 h after the last place trial on the 5th day of training with the platform being removed and the mouse being released from the quadrant opposite to where the platform had been located. Time spent in the various pool quadrants, including the target quadrant where the platform had been, and crossings over the exact platform location served as the dependent variables.

#### Holeboard exploration/olfactory preference test

Mice were evaluated for possible differences in exploratory behaviors and olfactory preference using a modified version of our previously published procedure [26], where hole poking served as the main behavioral response. Our protocol involved the use of a computerized holeboard apparatus (41×41×38.5 cm high clear plastic chamber), containing 4 corner and 4 side holes in the floor, with a side hole being equidistant between the corner holes (Learning Holeboard; MotorMonitor, Kinder Scientific, LLC, Poway, CA). Pairs of photocells were contained within each hole (27 mm in diameter) and were used to quantify the frequency and duration of pokes, whereby a poke that was at least 35 mm in depth was required to be registered as a hole poke. Odorants were placed at the bottom of two opposing corner holes, although access to the odorants was blocked. One corner hole contained a familiar odorant (fresh corn cob bedding), and the diagonally opposite corner hole contained a novel odorant (filter paper impregnated with 2 ml of coconut extract). The other pair of diagonally opposite holes was empty, as were all of the side holes. Holes containing odorants were counterbalanced between and within groups. Mice were administered a single 10-min trial, and general exploratory behavior was evaluated by quantifying total hole pokes, as well as pokes into the side and corner holes. Olfactory preference was assessed by analyzing poke frequencies involving odorant-containing versus empty corner holes, and novel versus familiar odorant-containing holes. Poke durations exhibited for the different types of holes were also analyzed to provide additional data on possible differences in the processing of olfactory stimuli.

#### Contextual fear conditioning test

Mice were evaluated on the conditioned fear test, as previously described [50]. The conditioned fear procedure was the last test conducted to avoid the possibility that exposure to footshock might affect subsequent behavioral performance. Briefly, the mice were trained and tested in two Plexiglas conditioning chambers (26 cm×18 cm, and 18 cm high) (Med-Associates, St. Albans, VT) with each chamber containing distinct and different visual, odor, and tactile cues. Each mouse was placed into the training/test chamber for a 5-min trial and freezing behavior was quantified during a 2-min baseline period. Over the next 3 min, the mice were exposed to 3 tone-shock pairings where each pairing included a 20-s presentation of an 80 dB tone (conditioned stimulus; CS) consisting of broadband white noise followed by a 1.0 mA continuous footshock (unconditioned stimulus; CS) presented during the last second of the tone. Broadband white noise was used instead of a frequency-specific tone in an effort to avoid possible auditory deficits that might occur with age. The mice were placed back into the conditioning chamber the following day and freezing behavior was quantified over an 8-min period to evaluate contextual fear conditioning. Twenty four hours later, the mice were placed into the other chamber containing different cues and freezing behavior was quantified during a 2-min “altered context” baseline and over the subsequent 8 min, during which time the auditory cue (tone; CS) was presented. Freezing was quantified using *FreezeFrame* image analysis software (Actimetrics, Evanston, IL), which allowed for simultaneous visualization of behavior while adjusting a “freezing threshold,” which categorized behavior as freezing or not freezing during 0.75 s intervals. Freezing was defined as no movement except for that associated with normal respiration, and the data were presented as percent of time spent freezing. Shock sensitivity was evaluated following completion of the conditioned fear testing, according to our previously described procedures [51].

### Histology

Animals were anesthetized and tissues were fixed by intracardiac perfusion with PBS followed by 4% PFA and post-fixation in 4% PFA. Paraffin sections were prepared with standard procedures and sagittal brain sections (10 µm) were obtained. It was Nissl stained, or immunostained with antibodies reactive against MAP2 (1∶500; Millipore), GFAP (1∶1000; Sigma), NeuN (1∶500; Chemicon) or Iba1 (1∶250; Wako) and incubated with DyLight549-conjugated secondary antibodies (Jackson ImmunoResearch, 1∶500) or stained with DAB. RBPj staining was performed as previously described [52] with some modification. Briefly, T-6709 antibody (Cosmo Bio, 1∶250) was incubated with brain section and the signal was amplified with ABC, TSA and detected with DAB.

Images were analyzed with Nikon Eclipse 80i, Apotome (Developmental Biology Histology Core at Washington University) or Nanozoomer (Alafi Neuroimaging Laboratory at Washington University).

### Stereology

Cells were counted by the optical dissector method using Stereo Investigator software (MBF Bioscience). Cortex was outlined using 4x objective and cell quantification was performed using 10x objective. Section interval was 5–8. The counting frame size was 40 µm×40 µm. The grid size was 300 µm×300 µm. The thickness of the tissue was 50 µm. These conditions were optimized to obtain a Gundersen coefficient of error less than 0.1.

### Data Analysis

Analysis of variance (ANOVA) models were used to analyze the behavioral data (Systat 12, Systat Software, Chicago, IL). Repeated measures (rm) ANOVA models containing two between-subjects variables (genotype and sex) and one within-subjects (repeated measures) variable (e.g., blocks of trials) were typically used to analyze the learning and memory data. The Huynh-Feldt adjustment of alpha levels was utilized for all within-subjects effects containing more than two levels to protect against violations of sphericity/compound symmetry assumptions underlying rmANOVA models. One-way ANOVA models were also used to analyze other data (e.g., 1-h locomotor activity test and measures in the sensorimotor battery). Planned comparisons were conducted within ANOVA models for certain holeboard test variables. In most other instances, pair-wise comparisons were conducted following relevant, significant overall ANOVA effects, which were subjected to Bonferroni correction when appropriate.

## Supporting Information

Figure S1 **Experimental design.** The same cohort of mice (control, RBPcKO het and RBPcKO homo) were subjected to behavioral then biochemical/immunohistochemical analyses. A separate cohort of mice were used for the qPCR studies.
(TIF)
