# Supplementary figures and images for: Loss of RBPj in Postnatal Excitatory Neurons Does Not Cause Neurodegeneration or Memory Impairments in Aged Mice
Source: PLoS One. 2012 Oct 26;7(10):e48180. doi: 10.1371/journal.pone.0048180 (PMC3482205; doi:10.1371/journal.pone.0048180)

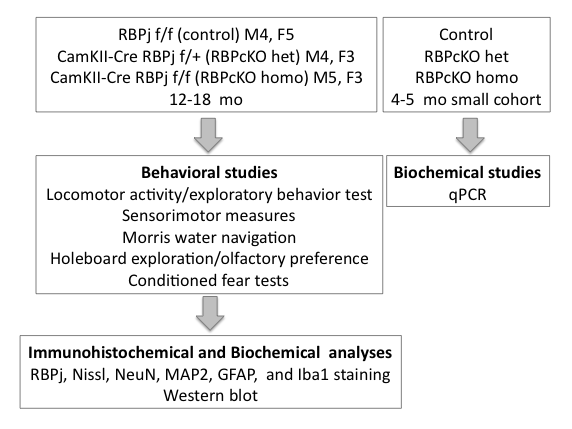

Supplement: Figure S1 — Experimental design. The same cohort of mice (control, RBPcKO het and RBPcKO homo) were subjected to behavioral then biochemical/immunohistochemical analyses. A separate cohort of mice were used for the qPCR studies. (TIF) [file pone.0048180.s001.tif]
